# Impact of Virtual Reality Intervention on Anxiety and Level of Cooperation in Children and Adolescents with Autism Spectrum Disorder during the Dental Examination

**DOI:** 10.3390/jcm13206093

**Published:** 2024-10-12

**Authors:** Abdulaziz Abdullah Al Kheraif, Tasneem Rashed Adam, Aisha Wasi, Raghad Khalid Alhassoun, Rawan Mohammed Haddadi, Mohammed Alnamlah

**Affiliations:** 1 Dental Health Department, College of Applied Medical Sciences, King Saud University, Riyadh 11433, Saudi Arabia; tasneemr.m94@gmail.com (T.R.A.); aishawasi98@hotmail.com (A.W.); raghadalhassoun11@gmail.com (R.K.A.); 2 Feedback Center, Princess Nourah Bint Abdulrahman University, Riyadh 11564, Saudi Arabia; rawan.hd@hotmail.com; 3 Department of Psychology, King Fahad Medical City, Riyadh 12231, Saudi Arabia; m.namlah@gmail.com

**Keywords:** autism spectrum disorder, dental anxiety, oral health, oral hygiene, virtual reality

## Abstract

**Background:** Individuals with Autism Spectrum Disorder (ASD) frequently encounter increased levels of anxiety and display resistant behaviors during dental examinations, which negatively affects their oral care and maintenance. This study employed a cross-sectional design to evaluate the impact of virtual reality (VR) intervention on the anxiety and level of cooperation in children and adolescents with ASD during dental examinations. **Methods:** A total of 140 participants diagnosed with ASD, aged from 4- to 18-years-old, were selected from two specialized ASD management centers in Riyadh/Saudi Arabia. The participants were randomly allocated into either the control group or the VR group. Control group participants were subjected to a conventional dental examination, while the VR group utilized VR intervention to immerse themselves in a simulated natural and soothing environment. The Venham anxiety and behavior scale (VABS) was utilized to measure anxiety levels, while the Frankl behavior rating scale (FBRS) was employed to assess the level of cooperation. Data were analyzed using a Mann–Whitney U test with a significance level of *p* < 0.05. **Results:** The baseline anxiety and level of cooperation between the groups were comparable (*p* > 0.05). During the dental examination, the VR group had significantly reduced anxiety scores (2.48 ± 1.76) compared to the control group (1.50 ± 1.74) (*p* < 0.001). Regarding the level of cooperation, the VR group exhibited significantly greater levels of cooperation (3.41 ± 0.96) than the control group (2.86 ± 1.03) (*p* = 0.002). **Conclusions:** These findings suggest that VR intervention is a successful technique for decreasing anxiety and enhancing cooperation among children with ASD during dental examination. Integrating VR technology in dental environments can potentially improve the dental experience and results for children diagnosed with ASD.

## 1. Introduction

Neurodevelopmental disorders in children and adolescents, such as autism spectrum disorder (ASD), intellectual disorder (ID), attention-deficit/hyperactivity disorder (ADHD), learning disability (LD), and others, can cause significant delays or abnormalities in growth, particularly in terms of functional, structural, and cognitive development [1,2,3]. Furthermore, it has been demonstrated that people who exhibit symptoms of anxiety and depression and people who have lived with individuals with an history of mental illness have a higher incidence of ADHD, ASD, ID, and LD [4].

Autism spectrum disorder refers to a continuum of early onset pervasive lifelong neurodevelopmental disorders portrayed by an inadequacy in social reciprocal interactions and communication, as well as a limited, repetitive pattern of interests, activities, and behavior [5]. According to recent estimates, the prevalence of ASD is currently considered to be 1 in 36 people [5].

Regarding the health implications of ASD on child populations, poor oral hygiene poses an unacceptable risk of developing oral diseases. As part of their routine oral hygiene practice, children with ASD frequently exhibit poor and irregular brushing habits and lack the physical dexterity necessary for good tooth brushing. Similarly, children with ASD have demonstrated a significantly higher caries prevalence index compared to children with normal development. This has been attributed to poor oral hygiene and a cariogenic diet. Firstly, children with autism have a preference for cariogenic diets, especially those high in sugar. Additionally, during specialized training and interviews, caregivers frequently employ cariogenic foods—such as candy and snacks—as an incentive to promote good behavior [6]. Secondly, children with ASD frequently retain food in their mouths for extended periods rather than swallowing it, due to their lack of tongue coordination, which also raises the risk of caries by exposing oral bacteria to sources of carbohydrates [7]. Lastly, the use of certain medicines for the treatment of ASD causes dry mouth (xerostomia), which is another reason that could account for the high rate of dental caries in children with ASD. Dry mouth may result from antidepressants, antipsychotic, and psychostimulant medications, which are the primary choice of treatment for autism [8].

Overall, poor oral health has a substantial negative influence on the quality of life of an individual with ASD [9]. As a result, it is crucial to incorporate regular dental examinations and subsequent dental procedures into the therapeutic regimens for individuals with autism [9]. The challenges of treating an ASD population are revealed in a recent analysis by Sami et al. [10]. This review emphasizes the high percentage of dental procedures performed under general anesthesia, which has raised concerns. These outcomes underline the critical need for customized approaches to oral healthcare, which necessitates practices that take into account the specific needs and behavioral characteristics of individuals with ASD. Research indicates that treating individuals with ASD might be challenging because of restricted capacity, capabilities, psychomotor skills, poor cooperation, issues with communication and behavior, and hypersensitivity to external stimuli [8,11]

Anxiety may be exacerbated in children diagnosed with ASD, which can have a significant impact on their dental care. Dental anxiety arises when individuals are exposed to the dental setting, leading to suboptimal outcomes in dental treatment and, ultimately, compromised oral health [12]. Additionally, resistive behaviors are frequently displayed by children with ASD during dental examinations. This hinders their oral hygiene maintenance and may be linked to anxiety related to dental visits as well as broader issues with oral hygiene [13,14]. According to a recent systematic review, lowering dental anxiety in children with ASD is essential for improving cooperation in a sensory-adapted dental environment [14]. The resistive behaviors and atypical reactions of patients with ASD is also due to heightened sensitivity to sensory stimuli, including sound, intense light, and touch, which are all present in a dental office [14]. These distressing stimuli could disrupt the therapeutic process [15]. Previous studies have reported that children diagnosed with ASD are frequently inclined to acquire information through visual stimuli, particularly electronic screen media like television, movies, and DVDs [16]. In that context, virtual reality (VR) intervention has become popular and is a novel distraction technique for reducing anxiety and behavior modification in pediatric patients [17].

Virtual reality is defined as an artificial environment experienced through sensory input, usually audiovisual, in which an individual’s actions have a partial impact on the environment [18]. Medical facilities commonly use VR intervention to distract patients’ attention during uncomfortable procedures like cast removal, vaccinations, and brief bedside procedures. It has been demonstrated to be effective in reducing procedure anxiety and improving patients’ quality of life [19]. Virtual reality is a kind of human–computer interaction that provides an enhanced degree of immersion by displaying incredibly lifelike digital images directly in the user’s field of vision. Virtual reality offers a better experience than traditional distraction approaches because it can maintain children’s attention and kindle their curiosity, making learning more enjoyable [20]. Also, children can practice social skills in a secure and regulated environment in VR without worrying about any potential negative consequences that could occur in the real world [21]. This could reduce anxiety and increase the motivation to participate [22]. In an systematic review by Alizadeh-Dizaj et al. [23], it was concluded that VR technologies improve the social and communication abilities of children with ASD and offer them an interesting and enjoyable experience. By using VR’s immersive and distraction features at the dental office, the dentist can assist autistic patients’ focus on a particular scenario and separate them from their surroundings, which will distract them from the dental environment, also known as sensory shielding [16].

In view of the available limited evidence, positive outcomes have been shown when VR was used to treat dental fear and anxiety (DFA) during dental treatment procedures [18,24,25,26,27]. Fakhruddin et al. [15] utilized audiovisual (AV) distraction using video eyewear to divert children’s attention from these distressing stimuli, thoughts, and emotions. According to the results of a recent clinical trial, VR effectively reduced young children’s DFA during dental treatments [25], and was favored over conventional behavior modification techniques for managing dental extraction-related anxiety in children [28]. Pagano et al. [8] demonstrates that patients with ASD could benefit from using VR to help them prepare for real dental visits. The effectiveness of VR distraction techniques in overcoming dental anxiety and behavior in healthy children is well documented [1,29,30,31,32]. However, studies evaluating the effectiveness of VR techniques on the anxiety and behavior in children and adolescents with ASD is scarce [33].

To the best of the authors’ knowledge, only one study by Suresh and George evaluated the effect of VR intervention on anxiety and behavior of children with ASD in a dental setting [16]. The authors reported that VR distraction was not just successful but was also a highly effective behavior management method for reducing the anxiety of autistic children during routine dental treatment. However, the outcome was limited by a low sample size (N-40) and the authors recommended further clinical studies and trials in different dental settings. In a recent systematic review, Cunnigham et al. [33] stated that VR is a promising tool in dentistry, but it has yet to be used to its full extent. The authors recommend high-quality trials and clinical studies to assess its use in dentistry, especially in cohorts of patients who may derive the most benefit, such as individuals with ASD.

Therefore, this study aimed to assess the effectiveness of VR as an audiovisual intervention technique on anxiety and the level of cooperation of 4–18-year-old children and adolescents with ASD during dental examinations using a well-established assessment instrument. If successful outcomes are observed, this concept can be applied in various medical and cultural settings, enhancing the oral health quality of life of those individuals with ASD. It was hypothesized that there would be no significant difference in the anxiety and level of cooperation of autistic children and adolescents with or without the use of VR intervention during dental examination.

## 2. Materials and Methods

### 2.1. Study Design and Centers

This study is a cross-sectional comparative study conducted at two centers. The Prince Nasser bin Abdulaziz Centre for Autism and the Riyadh Specialized Rehabilitation Center are state-run establishments overseen by the Ministry of Human Resources and Social Development in the city of Riyadh. Both centers are specifically committed to assisting individuals with ASD and ADHD, including both moderate and severe instances. Furthermore, the two centers have been operating and providing academic, social, and psychological services to individuals with special needs for past 25–27 years in accordance with international standards.

### 2.2. Participants Sampling and Selection

The participants’ inclusion criteria were as follows: (a) an age between 4 and 18 years, (b) a known diagnosis of ASD, (c) a demonstrated understanding and ability to comply with simple instructions, and (d) the signing of informed consent. Participants were excluded if they were blind or deaf, had undergone a recent eye infection or surgery, or were patients with a history of epilepsy, in order to reduce the likelihood of triggering epileptic seizures.

One hundred and forty participants aged between 4 and 18 participated in this comparative study. The sample size was based on effect size d = 0.5, α err prob = 0.05, power = 0.8, allocation ratio (N2/N1) = 1, and two-tailed (G* power), Version 3.1.9. 6 (Heinrich Heine University, Düsseldorf, Germany). The effect size (n1 = n2) was determined based on the pilot data of twenty participants with ASD which gave mean anxiety scores of 3.39 ± 0.89 for control and 2.96 ± 0.82 for intervention groups, respectively. A total sample of 128 participants was estimated; however, we recruited additional participants to compensate for any possible backout during the study (N = 140). The participants or the data from the preliminary study were not included in the final study or analysis.

### 2.3. Ethical Consideration, Informed Consent and Confidentiality

The present study was conducted from April to July 2024. The study protocol was consistent with the World Medical Association Declaration of Helsinki, adopted in 1964 and revised in 2013, for medical research involving human subjects. The study obtained ethical approval from the Institutional Review Board (IRB) at King Saud University (No-E-24-8445) in March 2024. Before conducting the study, the parents or caregivers of each participant provided their consent by signing the informed consent forms after being briefed about the purpose and protocol of the study. The participant’s parents or caregivers were assured of the confidentiality of the data, which were securely stored and ethically used and shared exclusively among the researchers.

### 2.4. Participants Grouping and Intervention

Using simple random sampling software (www.randomizer.org, accesses on 21 July 2024), the included participants, who were diagnosed with ASD and ADHD, were randomly assigned to either group 1 (control or conventional examination) or group 2 (VR intervention examination). The randomization and allocation concealment were performed by a dental hygienist who was unaware of the study process to prevent any bias. The study followed a single blinding process where the participants were not aware of their group. The prospective lack of VR glasses and the randomization of the participants were explained to the parents or caregivers. The research assistants gathered the participants’ demographic information such as age, sex, education, and year of enrollment at the center from their medical records before the clinical oral examination. After baseline data on anxiety and cooperation levels were collected using standardized assessment instruments, the clinical oral examination of the participants commenced.

The participants were required to visit the examination room only once during the study period. Each participant was led to the examination room by their caregiver and seated on an adjustable chair. The clinical examination was conducted utilizing disposable dental examination kits under natural light in the presence of their caregivers. All of the clinical examinations were performed by qualified and calibrated dentists (A. I. and R. K. A). The participants were instructed to split their lips, and during this period, their willingness to comply was determined. The dentists performed the dental examination once the participant consented to open his/her mouth.

**Figure 1:**
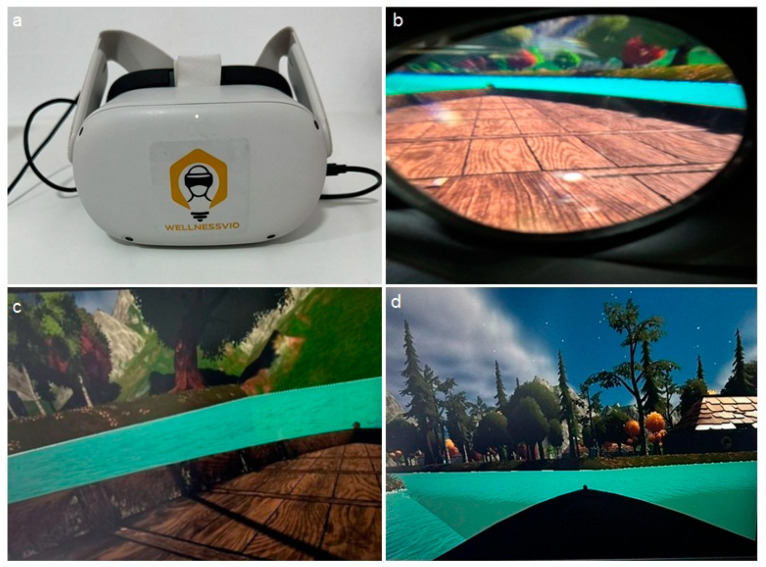
Virtual reality intervention: (**a**) Oculus Quest 2 VR headset; (**b**) the customized interactive environment as viewed from the VR headset; (**c**,**d**) still images of the navigation through the interactive environment.

The participants in group 2 underwent the same examination process but with the VR technology intervention. The VR headset was pilot tested on the ten participants for acceptance and reliability and the ability of the participants to follow simple instructions. Each participant was introduced to the VR eyewear using the tell–show–do method. They were given the VR headset and permission to use it, allowing them to recognize and familiarize themselves with the headset. Before oral examination, each patient had the VR headset placed on their head, their consent was verified, and they were asked if the headset caused pain or discomfort. The participants were allowed to experience the VR content for 2–3 min before the oral examination. The VR intervention was conveyed utilizing an Oculus Quest 2 VR headset (Meta Platforms, Inc., Menlo Park, CA, USA) (Figure 1a) and a customized software program developed by WellnessVio-VR (Al-Ulaya, Riyadh, Saudi Arabia), a Riyadh-based firm. Wellnessvio-VR designed a customized interactive environment to ensure that the participants remained engaged by placing the individual on a boat and navigating through a deep forest along a river. Furthermore, the VR environment utilizes a simulated natural setting with aquatic ecosystems, such as rivers and wetlands, by integrating cartoon characters and soothing musical tones to offer a calming and tranquil experience (Figure 1b–d). It was designed with a primary emphasis on achieving maximum relaxation.

During the oral examination, psychologists closely monitored the participants and documented the anxiety and level of cooperation. A clinical record file was used to record the oral health status and the examination findings, and the complete dental examination and intervention was limited to seven to ten minutes.

### 2.5. Data Collection and Assessment Tools

This study included diverse datasets to offer a thorough analysis by compiling information on demographic characteristics, such as age and gender, baseline anxiety and behavior levels, and relevant clinical records. Both digital and manual record-keeping techniques were implemented to ensure accuracy. The well-established VABS and FBRS instruments were used to evaluate the participants’ anxiety and level of cooperation.

#### 2.5.1. Venham Anxiety and Behavior Scale (VABS)

The Venham anxiety and behavior scale (VABS) is a comprehensive assessment instrument used to assess children’s anxiety and behavior during dental visits. The assessment thoroughly assesses a child’s anxiety and behavioral state throughout a dental procedure by integrating elements from VABS and independent behavioral observations [34]. A VABS with a range of 0 to 5 is used to rate the anxiety and behavior. Each level corresponds to a rise in observable anxiety (Table 1).

#### 2.5.2. Frankl Behavior Rating Scale (FBRS)

In clinical dentistry and research, particularly pediatric dentistry, the Frankl behavior rating scale (FBRS) is a widely used behavior rating system for assessing children’s general behavior and level of cooperation during dental visits. The physician can gather treatment options using this instrument [34]. Table 2 outlines the 4-point FBRS scoring description used to evaluate behavior on a scale of 1 to 4.

These evaluations, conducted by competent psychologists (R.M.H. and M.A.), improve overall safety observation by thoroughly understanding how children respond to the interventions. Scores were assigned to each participant to allow subsequent analysis.

### 2.6. Statistical Analysis

The data were analyzed using the Statistical Package for Social Sciences [SPSS] for Windows V. 22, released in 2013 (IBM Corp., Armonk, NY, USA). The descriptive analysis of the explanatory variables is presented as mean and standard deviation (SD) for continuous variables and as frequency and proportions for categorical variables. Gender distribution was compared using the Chi square test. The Shapiro–Wilk and Kolmogorov–Smirnov tests revealed that the data did not follow a normal distribution. The Mann–Whitney U test, a non-parametric test used to investigate differences between two groups when the dependent variable is ordinal and does not follow a normal distribution, was employed in the statistical study. The mean and SD values of the age distribution and anxiety and level of cooperation scores between the control and VR groups were analyzed using the Mann–Whitney U test. A *p*-value ≤ 0.05 was regarded as statistically significant.

## 3. Results

This study assessed and compared the anxiety and level of cooperation of children and adolescents during dental examinations with or without VR interventions. The participant’s anxiety and level of cooperation was assessed utilizing VABS and FBRS instruments, respectively. There were no dropouts and all the 140 participants recruited for this study were available for final analysis.

### 3.1. Study Population

The study population included 140 children and adolescents: 70 (50%) in the control group and 70 (50%) in the VR group. The mean age of the participants was 11.77 ± 3.44 years. The control group had a mean age of 10.62 ± 2.51 years, while the VR group had a mean age of 12.1 ± 2.60 years, indicating a non-significant difference between the groups (*p* < 0.05).

Regarding gender, 106 males (74.9%) and 34 females (25.1%) participated in this study. Within the control group, 55 (78.6%) were male and 15 (21.4%) female, while in the VR group, 19 were females (27.1%) and 51 were males (72.9%), and the gender distribution was comparable between the groups (Table 3).

Among the participants, 136 (97.2%) were diagnosed solely with ASD, while the remaining 4 (2.8%) were diagnosed with both ASD and ADHD.

### 3.2. Baseline Anxiety and Level of Cooperation between the Groups

All the participants, irrespective of the study groups, were subjected to baseline anxiety and level of cooperation; the scores are presented in Table 4. The mean VABS scores of the control and VR groups were 3.80 ± 1.27 and 3.78 ± 0.88, respectively, with no significant difference between the scores (*p* = 0.430) (Table 4). Similarly, the median FBRS scores for the control and VR groups were 1.77 ± 0.75 and 1.85 ± 0.81, with no significant variations between the groups (*p* = 0.651) (Table 4). Thus, the study groups were comparable in terms of the baseline anxiety and level of cooperation scores.

### 3.3. Anxiety and Level of Cooperation between the Study Groups

**Figure 2:**
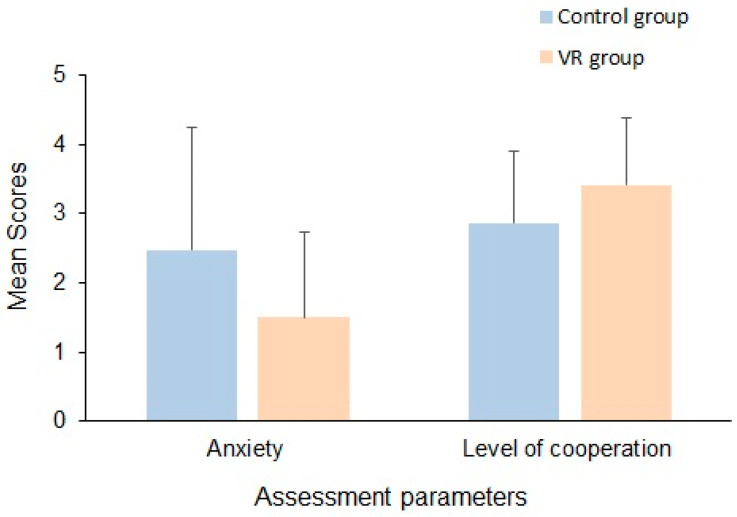
Mean VABS and FBRS scores indicating the anxiety and level of cooperation of the study groups during dental examination.

The mean scores of anxiety and level of cooperation of the study groups during dental examination are presented in Figure 2. The anxiety score as assessed using the VABS was 2.48 ± 1.76 for the control group, while it was 1.50 ± 1.24 for the VR group. The Mann–Whitney U test indicated a significant difference between the two groups (*p* = 0.001), suggesting that the participants in the VR group displayed less anxiety than the control group during dental examinations (Table 5).

Comparing the level of cooperation between the control and VR groups, as evaluated using the FBRS, the control group exhibited a mean score of 2.86 ± 1.03, while the VR group demonstrated a mean score of 3.41 ± 0.96. The Mann–Whitney U test revealed a significant difference between the study groups (*p* = 0.002), indicating that the participants in the VR group showed higher levels of cooperation than the control group during dental examinations (Table 5).

### 3.4. Anxiety and Level of Cooperation between the Study Groups Quantified by Age

The comparison of anxiety and level of cooperation between the control and VR groups, quantified by age (children ≤ 12 years; adolescents > 12 years), is presented in Table 6. Significant differences in anxiety and cooperation levels were observed based on age group, with children showing higher anxiety and less cooperation in the control group compared to the VR group. Regarding anxiety, there was a significant variation between the groups, with the control group demonstrating higher mean scores (2.71 ± 1.65) compared to the VR group (1.54 ± 1.81) (*p* = 0.019). Also, adolescents showed a significant variation in mean anxiety scores (2.42 ± 1.80) compared to the VR group (1.17 ± 0.98) (*p* = 0.013) (Table 6).

Regarding the level of cooperation, there was a significant difference between the groups, with the control group demonstrating higher mean scores (2.82 ± 1.02) compared to the VR group (*p* = 0.014). For adolescents, there was no significant difference in the level of cooperation between the groups (*p* = 0.118) (Table 6).

## 4. Discussion

The presence of anxiety during dental examinations may cause individuals with ASD to display greater resistance and discomfort. This could make it more challenging for them to receive necessary dental care and have productive conversations with the dentist effectively. In the current study, it was hypothesized that relative to the conventional dental examination method, the VR simulation would have a positive impact on the anxiety and level of cooperation of children and adolescents with ASD. Based on the data analysis outcomes, the study hypothesis was partially rejected. The VR intervention was effective in reducing anxiety and enhancing the level of cooperation in children, but it did not have any significant effect on enhancing the level of cooperation among the adolescents.

Individuals diagnosed with ASD sometimes struggle to have confidence in the professional’s competence to deliver exceptional jobs, which may be linked to deficiencies in mentalizing, a fundamental characteristic of ASD [35]. Dentists utilize many techniques to address the needs of patients who suffer from fear or anxiety. According to Gurav et al. [36] and Lu et al. [37], the top two intervention techniques for reducing dental anxiety are music and VR, respectively. VR distraction is a noninvasive and safe method that does not require any prior knowledge or expertise. It provides a pleasant experience that effectively reduces unpleasant feelings, such as anxiety, by distracting the user’s attention from the dental environment that might cause anxiety [38]. VR distraction shows a significant potential in mitigating discomfort and anxiety associated with various dental procedures. Its long-term benefits include patients’ increased willingness to return for treatment, giving them a more positive memory of their sessions [39]. The current study employed interactive games and 3D virtual simulations of real-life scenarios as stimuli for VR distraction that provide a high degree of ecological validity. This technique can be effectively utilized in children and adults by tailoring the visual content to align with their stage of development [40]. Furthermore, the customized software can be more visually appealing than regular video, maximizing the benefits of VR. Children can also receive positive reinforcement in the form of digital content and more detailed information about the treatment in an appropriate manner [28].

In this study, two reliable and validated assessment instruments, the Venham anxiety and behavior scale (VABS) and the Frankel behavior rating scale (FBRS), were used. The VABS is a straightforward instrument for measuring the anxiety and uncooperative conduct of children in a dental setting. The five behaviorally defined categories on the scale range from 0 to 5, with a higher score denoting a higher degree of anxiety. This scale is highly reliable, even for inexperienced observers [41]. Furthermore, this scale is validated by comparing the anxiety and uncooperative behavior ratings with a number of self-report and physiological indices of how children react to dental treatment. The results show a strong association, confirming the validity of the rating method [41]. One of the most practical behavior evaluation instruments in pediatric dentistry research and routine clinical practice is the FBRS, developed in 1962. Depending on the child’s attitude during dental treatment, behavior can be classified into four categories—certainly positive to definitely negative—that can be used at different treatment points. It is regarded as one of the most reliable instruments for rating children’s compliance in dental settings, making it an indispensable instrument in routine practice [42,43]. Furthermore, FBRS is a validated tool with high inter-examiner reliability [43].

The control group, in this study, demonstrated increased anxiety, which was further intensified by their challenges in comprehending the dentist’s goals and effectively handling sensory stimuli and expectations [35]. On the contrary, children in the VR group had significantly reduced anxiety scores during dental examination. This is consistent with the study by Aminabadi et al. [29], who observed a notable decrease in anxiety by employing VR intervention in dental environments for children between 4- and 6-years-old. According to the study results by Fakhruddin and El Batawi [15], the use of video eyewear was a significant intervention for 6.5–9.8-year-old children with ASD, effectively reducing their anxiety during noninvasive preventive dental procedures. When these children viewed cartoons on VR glasses instead of ceiling projections while receiving sealants or restorations, the authors observed a substantial decrease in anxiety, as indicated by a reduction in heart rate. These findings support our hypothesis that VR can significantly reduce anxiety compared to conventional methods. This also emphasizes VR’s effectiveness in managing anxiety in 4- to 18-year-old children during dental examinations.

Patient compliance can be defined as the extent to which an individual’s actions correspond with recommendations regarding oral health. It indicates a patient’s readiness to follow treatment or preventive recommendations provided by their dental professional. Patient compliance is essential for the long-term success of therapy [44]. This is particularly crucial for patients with ASD due to their poor cooperation and issues with communication and behavior [8,11]. In this study, it was observed that the VR group exhibited greater compliance during dental examinations compared to the control group (*p* = 0.002). These findings support our hypothesis that VR can significantly enhance patient compliance compared to conventional methods. Furthermore, the outcome of this study reiterates that dentists should consider using audio and VR intervention strategies in dental clinics to alleviate anxiety in children with ASD who are undergoing dental examination, while also improving their cooperative behavior. The enhancement of behavior is valuable not only for the child’s well-being, but also for the clinicians’ confidence in the accuracy and dependability of their examination, diagnosis, and subsequent treatment [14,33]. Previous studies have documented a range of non-compliant behaviors exhibited by autistic children during dental treatment [14]. This can be attributed to various factors, such as difficulties in communicating, disturbances in the child’s regular routines, increased sensitivity to sensory stimuli, and dread or anxiety associated with dental experiences [14,16,45]. These numerous influences could have a substantial impact on children’s attitudes and willingness to cooperate. There exists a correlation between dental anxiety and non-compliant conduct among children and adolescents, as high levels of dental anxiety are frequently associated with uncooperative behavior in children [46].

Adolescence—the stage of life in which youth aged 12 to 17 mature into young adults—is frequently disregarded in activities aimed at studying, evaluating, and improving oral health, with more emphasis on younger children and adults. Significant physical and neurological changes occur in adolescents, some of which may immediately impact dental health [47]. The anxiety and level of cooperation was further quantified according to the age of children (≤12 years) and adolescents (>12 years). The anxiety scores were significant in both VR age groups. On the contrary, we found a non-significant difference with the level of cooperation in the adolescent VR groups compared to the conventional groups (*p* = 0.118) This indicates that VR intervention was not effective with regard to the level of cooperation in the adolescent group. The difference in the outcome between these two age groups could be possibly related to the content of the VR intervention. The VR video, which was a good method to reduce anxiety and increase the level of cooperation in children, might not be suitable to enhance the cooperation of the adolescents. Therefore, it is necessary to adjust the VR content according to age, or it is recommended that patients be given the option of selecting the content themselves to achieve better results. This outcome was partially in agreement with the outcome of a study by Ghadimi et al. [48]. However, the previous studies were based on healthy children. It is important to note that dental anxiety and level of cooperation are two distinct conditions [48]. Klingberg and Broberg [49] found that while compliance issues were linked to activity and impulsivity, dental anxiety was more strongly associated with temperamental traits like negative emotions, inhibition, and shyness.

The current study has a few limitations. The study reported immediate outcomes focused on children and adolescents with autism in general, without any emphasis on different levels or severity of autism. The data based on the severity of autism could provide specific outcomes and a broader understanding of the anxiety and compliance levels of individuals as related to the severity of autism. The study used simple random sampling, demonstrating limited flexibility and large sample size variations which could have affected the outcome of the study. The study reported anxiety and level of cooperation during dental examination, but no individual oral and dental findings are reported. The individual dental findings could have provided information regarding the participant’s oral health status and treatment needs. The majority of the participants in this study were male, which is consistent with a higher prevalence of ASD in males. This presents another limitation of our study. The participants were classified based on two age groups, but the inclusion of a diverse population and different age groups could provide a broader understanding of the anxiety and level of cooperation in these groups of patients. Finally, there was no adjustment or modification of the VR content based on age and this could have contributed to the variations in the outcome of the study concerning adolescent participants.

The outcome of the study highlights the urgent need for more clinical trials and inclusive research samples with diverse backgrounds. Future studies should, therefore, prioritize the exploration of how different levels of autism and VR intervention affect the anxiety and level of cooperation of children and adolescents during dental examinations. More research is needed to include significant number of female participants to determine whether they exhibit similar trends related to anxiety and level of cooperation with the use of VR intervention compared to their male counterparts. It would be interesting to evaluate the adjustment or modification of VR content in accordance with patient’s age to assess the effect of VR content modifications on the anxiety and behavior of the participants in different age groups. Furthermore, the individual dental findings including dental caries, oral hygiene, and periodontal status should be recorded to evaluate the treatment needs of these special needs patient groups. It would be beneficial to discuss the potential long-term effects of VR intervention on dental care compliance and overall anxiety management. Finally, the effect of VR intervention during the dental treatment stages of autistic children and adolescents should be a key focus of future studies, as these directions hold significant potential for advancing our understanding and improving dental care for individuals with autism.

## 5. Conclusions

Within the limitations of this comparative study, it can be concluded that VR intervention is a successful technique for reducing anxiety and enhancing cooperation among children diagnosed with ASD during dental examination. Integrating VR technology in dental environments can potentially improve the dental experience outcome for children diagnosed with ASD. Further studies are needed to explore the long-term effects of VR interventions and their applicability across diverse patient populations.

Dentists should consider utilizing VR technology in their routine clinical practice for the treatment of patients with ASD, although it is recommended that the VR content be modified or adjusted in consideration of the age of the patients.

## Figures and Tables

**Table 1:** Rating and description of Venham anxiety and behavior scale (VABS).

Rating	Description
0	Relaxed, without any indication of anxiety
1	A little anxious, but readily comforted
2	Anxious, with sporadic displays of discomfort
3	Often exhibiting signs of discomfort or anxiety, but not overly anxious
4	Extremely tense, persistently uncomfortable behaviors, sobbing, or vocal objections
5	Extreme anxiety, panic attacks, strong opposition, or denial

**Table 2:** Rating and description of Frankl behavior rating scale.

Rating	Attitude	Description
1	Definitely Negative	Refusal to receive therapy, sobbing loudly, feeling frightened, or displaying any other obvious signs of extreme negativity
2	Negative	Reluctance to comply with treatment, uncooperative conduct, and mildly negative emotions (e.g., withdrawn, gloomy)
3	Positive	Acceptance of treatment, occasionally cautious demeanor, and readiness to follow the dentist’s instructions, albeit with a small amount of reluctance or anxiety
4	Definitely Positive	Showing a pleasant relationship with the dentist, expressing curiosity about the dental treatments, and being able to laugh and take pleasure in the moment

**Table 3:** Study population (n = 140).

		Study Groups		
		Control Group	VR Group	*p* Value
Age				
Age (years), Mean ± SD		10.62 ± 2.51	12.1 ± 2.60	*p* = 0.09 ^a^
	Gender			
Male, n (%)		55 (78.6%)	51 (72.9%)	*p* = 0.24 ^b^
Female, n (%)		15 (21.4%)	19 (27.1%)	

Statistically non-significant (*p* > 0.05); ^a^ Mann–Whitney U test and ^b^ Chi square test.

**Table 4:** Comparison of baseline anxiety and level of cooperation of the study groups.

Parameters	Study Groups		*p* Value
	Control Group	VR Group	
Anxiety	3.80 ± 1.27	3.78 ± 0.88	*p* = 0.430
Level of cooperation	1.77± 0.75	1.85 ± 0.81	*p* = 0.651

Statistically non-significant (*p* > 0.05; Mann–Whitney U test).

**Table 5:** Comparison of anxiety and level of cooperation of the study groups during the dental examination.

Parameters	Study Groups		*p* Value
	Control Group	VR Group	
Anxiety	2.48 ± 1.76	1.50 ± 1.24	*p* = 0.001 *
Level of cooperation	2.86 ± 1.03	3.41 ± 0.96	*p* = 0.002 *

* Statistically significant (*p* < 0.05; Mann–Whitney U test).

**Table 6:** Comparison of anxiety and level of cooperation of the study groups quantified by age.

Parameters		Study Groups		*p* Value
	Age	Control Group	VR Group	
Anxiety	≤12 years	2.71 ± 1.65	1.54 ± 1.81	*p* = 0.019 *
	>12 years	2.42 ± 1.80	1.17 ± 0.98	*p* = 0.013 *
Level of cooperation	≤12 years	2.82 ± 1.02	3.40 ± 0.97	*p* = 0.014 *
	>12 years	2.86 ± 1.04	3.30 ± 0.84	*p* = 0.118

* Statistically significant (*p* < 0.05; Mann–Whitney U test).

## Data Availability

The data available are presented in this study.
